# Supplementary material for: Histone variant H2A.Z regulates zygotic genome activation
Source: Nat Commun. 2021 Dec 1;12:7002. doi: 10.1038/s41467-021-27125-7 (PMC8636486; doi:10.1038/s41467-021-27125-7)
Supplement: Supplementary file 1 — Supplementary information [file 41467_2021_27125_MOESM1_ESM.pdf]

Histone variant H2A.Z regulates zygotic genome activation
Supplementary Information

Dafne Ibarra-Morales, Michael Rauer, Piergiuseppe Quarato, Leily Rabbani, Fides Zenk, Mariana Schulte-Sasse, Francesco Cardamone, Alejandro Gomez-Auli, Germano Cecere, Nicola Iovino

---

This supplementary file comprises 4 Supplementary Figures, 1 Supplementary Table, Supplementary Methods and Supplementary References.

Supplementary Figure 1
Chromatin states of transcriptionally active and inactive genes at ZGA, related to Fig.1

Supplementary Figure 2
Domino is the main H2A.Z chaperone at TSS during early embryogenesis, related to Fig.2

Supplementary Figure 3
H2A.Z depletion on promoter regions affects transcriptional activation at ZGA, related to Fig.3

Supplementary Figure 4
Domino depletion does not affect nucleosome positioning but alters local chromatin structure, related to Fig.4

Supplementary Table 1
List of antibodies used for this study

Supplementary Methods

Supplementary References

Supplementary Figure 1

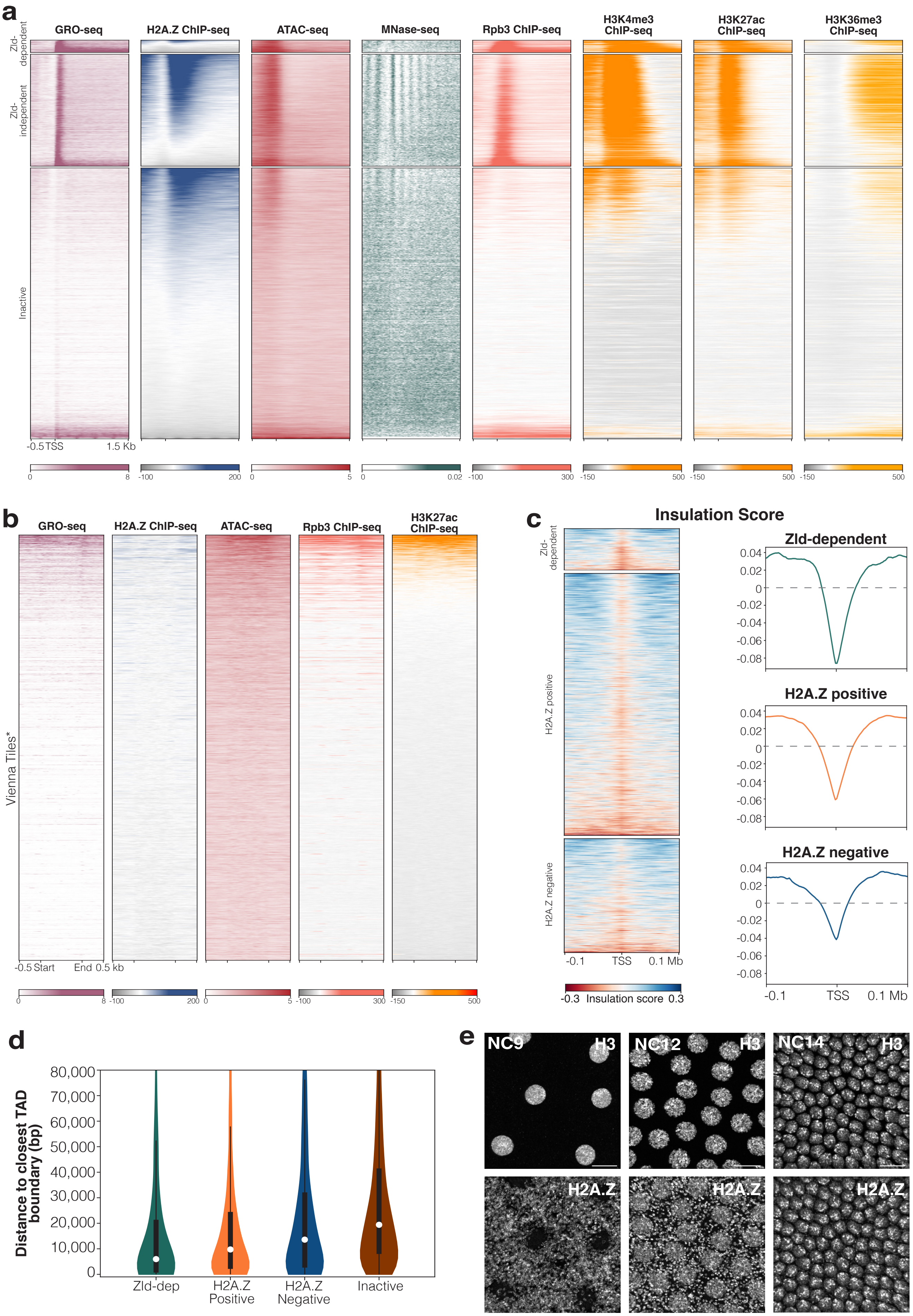

Supplementary Figure 1

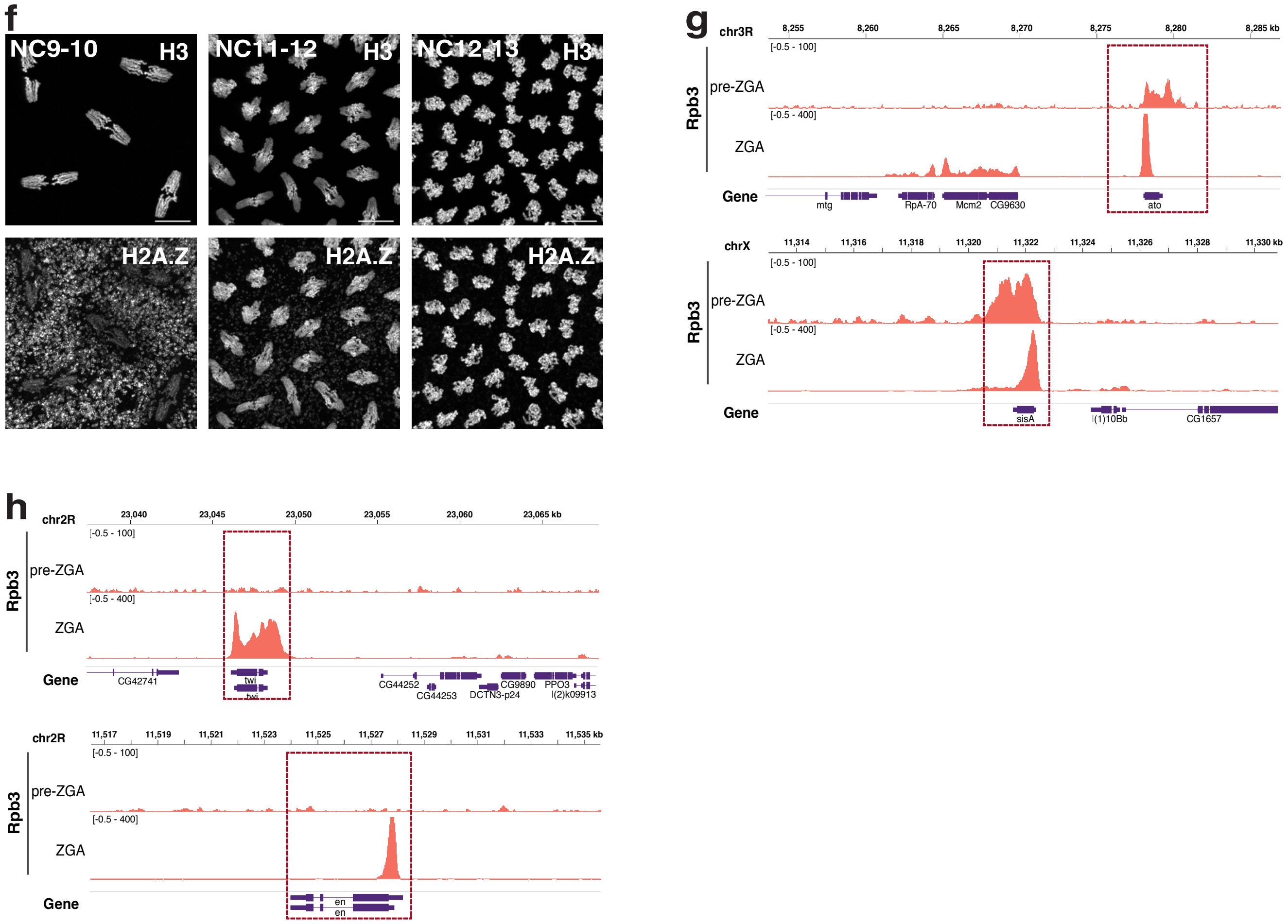

Supplementary Figure 1| Chromatin states of transcriptionally active and inactive genes at ZGA, related to Fig.1. a, Heatmap of all unique promoters in the Drosophila genome (see Methods), classified by transcriptional activity determined by GRO-seq signal, and sorted by H2A.Z ChIP-seq Ctrl signal. Active genes are further subdivided in Zld-dependent and Zld-independent as in Fig. 1. Different chromatin marks and features explored are named on top of each column. Rpb3 signal represents RNA Pol II binding. All signal tracks correspond to embryos at ZGA (NC14). **b**, Heatmap centered on the scaled Vienna tiles collection[1]. All signal tracks correspond to embryos at ZGA. No enrichment for H2A.Z is detected. **c**, Left: Heatmap of the insulation score, centered on TSS, of unique promoters grouped in Zld-dependent, H2A.Z positive and H2A.Z negative in control embryos at ZGA. Right: Profile plots of the same insulation scores. **d**, Distance of unique promoters of each group to the closest TAD boundary. Box plot inside depict the median (white dot) and the interquartile range (IQR) from the 1st to the 3rd quartile. Whiskers indicate the upper (Q3 + 1.5*IQR) and lower edge (Q1 - 1.5*IQR). n=2 biologically independent experiments. **e**, Immunostaining of nuclei from embryos at NC9 (left), NC12 (middle) and NC14 (ZGA, right). Before ZGA, H2A.Z can be seen both inside and outside the nucleus. The cytoplasmic staining in small rings correspond to lipid droplets, as reported before[2]. Scale bar = 10µm. Images are Z-projections. **f**, Immunostaining of mitotic nuclei from embryos NC9-NC10 (left), NC11-NC12 (middle), NC12-NC13 (right), showing that H2A.Z remains attached to the chromatin during mitosis. Scale bar = 10µm. Images are Z-projections. (**e, f**) Similar results were observed for at least 3 embryos of each stage. **g**, Screenshots of genome browser tracks (Integrative Genomics Viewer, IGV[3]) of Rpb3 signal (representing RNA Pol II binding) at two different stages: pre-ZGA and ZGA. ato (atonal) and sisA (sisterless A), two genes transcriptionally activated in the minor wave of ZGA are highlighted. **h**, Same as (**g**) but for twi (twist) and en (engrailed), two genes transcriptionally activated at ZGA, but not at pre-ZGA.

Supplementary Figure 2

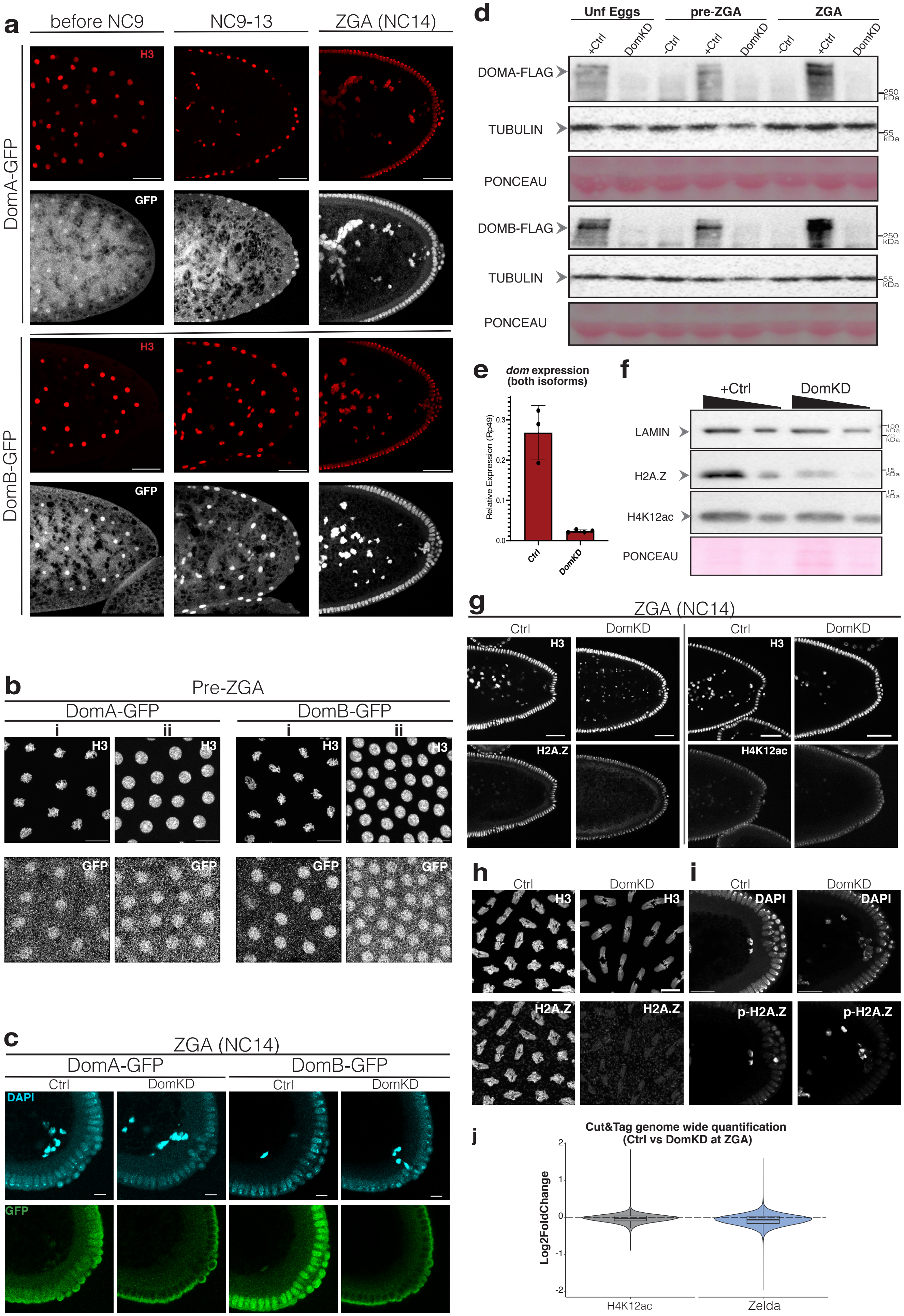

Supplementary Figure 2

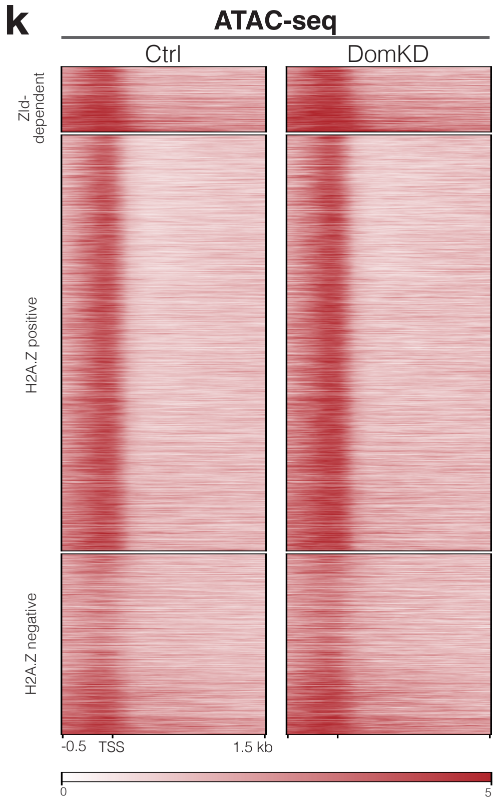

Supplementary Figure 2| Domino is the main H2A.Z chaperone at TSS during early embryogenesis, related to Fig.2. a, Representative immunostaining of DominoA-GFP-Flag (top) and DominoB-GFP-Flag (bottom) expressing embryos before NC9, at NC9-13 and at ZGA. H3 stains chromatin. Scale bar = 50µm. **b**, Representative immunostaining on nuclei of pre-ZGA (NC9-13) DominoA-GFP-Flag (left) and DominoB-GFP-Flag (right) expressing embryos. (i) During mitosis and (ii) during interphase. The cytoplasmic staining does not resemble the small ring structures seen for H2A.Z on lipid droplets (compare to Extended Data Fig. 1e, f). Scale bar = 15µm. **c**, Immunostaining of DominoA-GFP-Flag (left) and DominoB-GFP-Flag (right) in Ctrl and DomKD embryos showing that the shRNA used for DomKD depletes both Domino isoforms. Leftover green staining is background signal. Scale bar = 10µm. **d**, Western Blots of DominoA-GFP-Flag (top) and DominoB-GFP-Flag (bottom) in Ctrl and DomKD samples of unfertilized eggs and embryos at pre-ZGA and ZGA stages. Tubulin and Ponceau staining are showed as loading controls. 24 unfertilized eggs or embryos were loaded per lane. **e**, Relative expression of dom mRNA in ZGA embryos by qPCR for Ctrl and DomKD. n=3 for Ctrl and n= 4 for DomKD biologically independent samples. Data are presented as mean values +/- SD. Expression is shown relative to rp49. **f**, Western Blot of the chromatin fraction of Ctrl and DomKD 0-4hrs embryos. Lamin and Ponceau staining are showed as loading controls. 9µg and 3µg of extract were loaded per lane. (**d, e, f**) Source data are provided as a Source Data file. **g**, Immunostaining of Ctrl and DomKD embryos for H2A.Z (left) and H4K12ac (right) at ZGA. H3 stains chromatin. Scale bar = 50µm. (**a, b, g**) Images are Z-projections. **h**, Close up on nuclei of immunostained pre-ZGA embryos (NC9-13) in Ctrl and DomKD. H3 stains chromatin. Scale bar = 10µm. Images are Z-projections. **i**, Immunostaining of Ctrl and DomKD embryos for phospho-H2A.Z (p-H2A.Z) at ZGA. DAPI stains DNA. Scale bar = 25µm. **j**, Violin plot showing the log2 fold change between Ctrl and DomKD ZGA embryos obtained by Cut&Tag (spike-in normalized, see Methods for details) of H4K12ac on chromatin (left) and Zelda binding (right). Sites are restricted to canonical chromosomes (2L/R, 3L/R, 4, X). For both plots, there is no global shift, since the log2FoldChange distribution is centered around 0. Box plot inside depict the median and the interquartile range (IQR) from the 1st to the 3rd quartile. Whiskers indicate the upper (Q3 + 1.5*IQR) and lower edge (Q1 - 1.5*IQR). n=2 biologically independent experiments per genotype. k, Heatmap of ATAC-seq signal in Ctrl and DomKD embryos at ZGA, centered on TSS.

Supplementary Figure 3

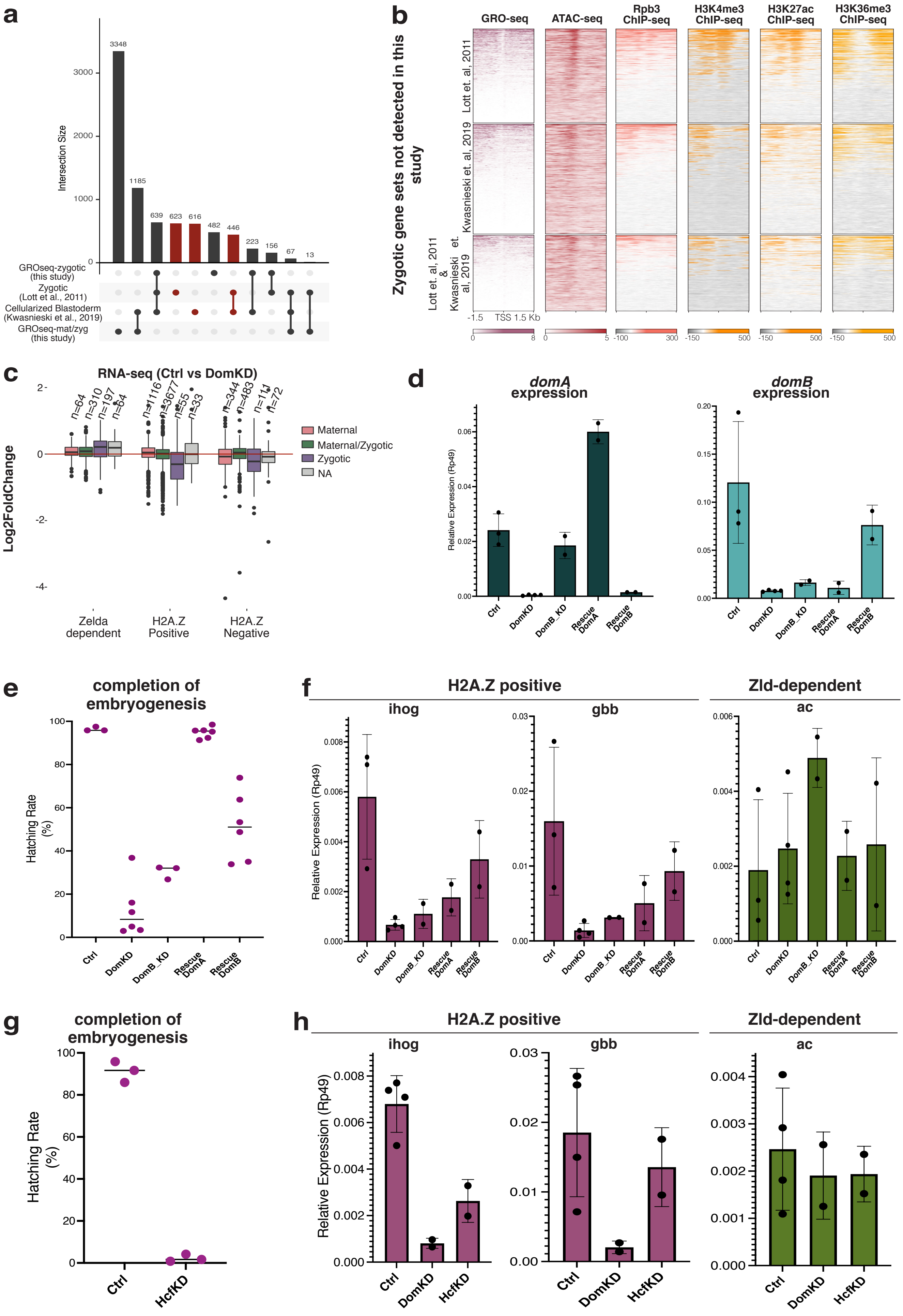

Supplementary Figure 3

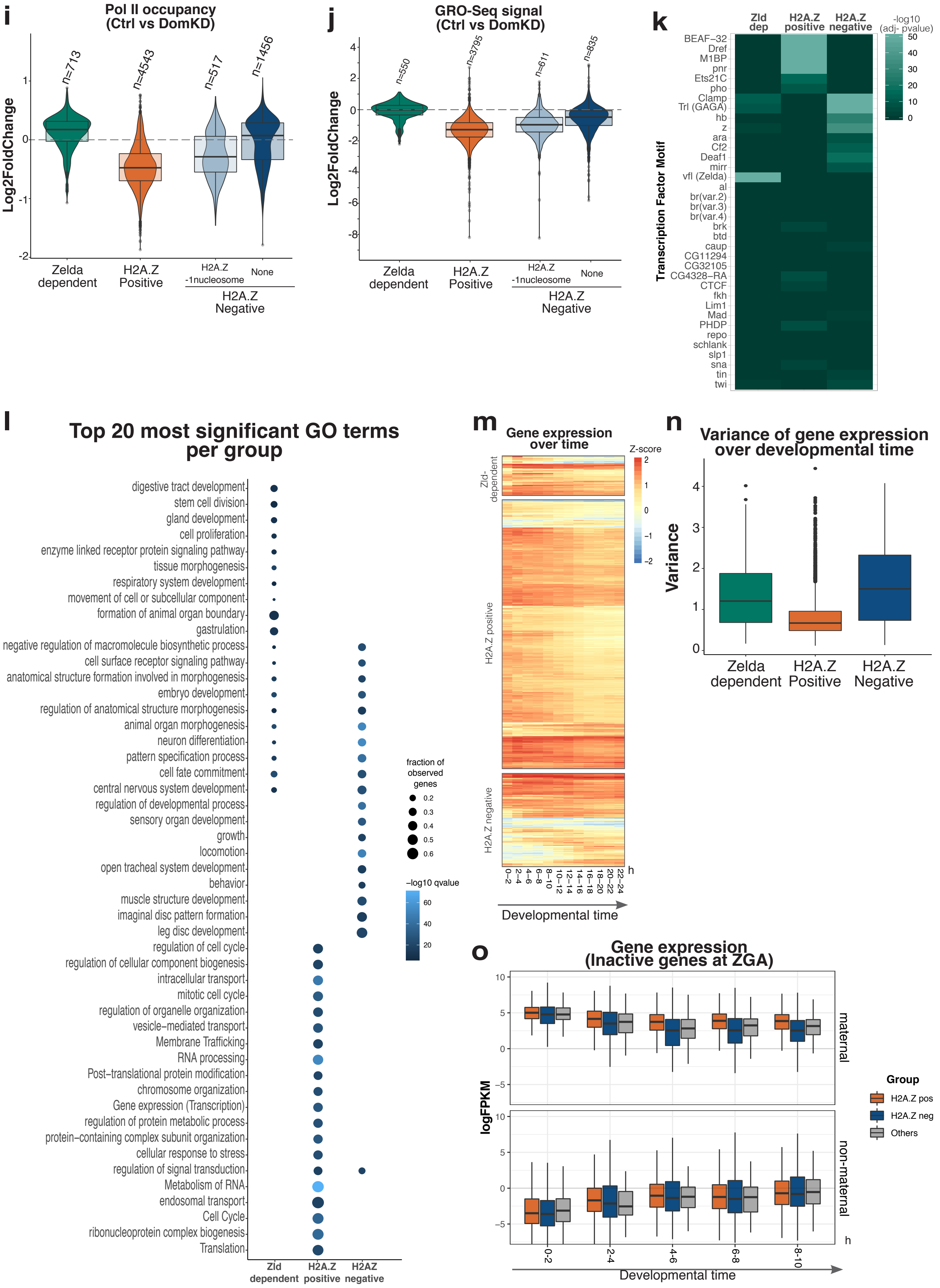

Supplementary Figure 3| H2A.Z depletion on promoter regions affects transcriptional activation at ZGA, related to Fig.3. a, Upset plot of the pure zygotic (GROSeq-zygotic) and maternal/zygotic (GROSeq-maternal/zygotic) genes found in this study compared to the zygotic genes found in Lot et al., 2011[4], and the maternal/zygotic and zygotic genes found by Kwasnieski et al., 2019[5]. Groups containing genes not found in this study are highlighted in red. The number of genes on each group or intersection is depicted at the top of each bar. **b**, Heatmap centered on TSS of zygotic genes not found to be expressed at ZGA by GRO-seq in this study. The signal of GRO-seq, ATAC-seq, RNA Pol II (Rpb3 ChIP-seq) and several histone activation marks is plotted. All signal tracks correspond to embryos at ZGA (NC14). **c**, Box plot of the log2FoldChange in transcript levels (RNA-seq) of ZGA embryos in the Zld-dependent, H2A.Z positive and H2A.Z negative genes classified by origin in maternal, maternal/zygotic and zygotic genes. Only genes with TPM > 5 in Ctrl or DomKD embryos were retained. NA = not assigned. **d**, dominoA (domA, left) and dominoB (domB, right) mRNA expression in ZGA embryos of Ctrl, DomKD, DominoB only knockdown (DomB_KD), rescue by DomA overexpression (Rescue DomA), or rescue by DomB overexpression (Rescue DomB), as measured by qPCR. **e**, Percentage of embryos completing embryogenesis (Hatching rate) in different conditions (Ctrl, DomKD, DomB_KD, Rescue DomA, Rescue DomB). **f**, Expression of two H2A.Z positive genes (left and middle) and a Zld-dependent gene (right) in different conditions (Ctrl, DomKD, DomB_KD, Rescue DomA, Rescue DomB) as measured by qPCR on ZGA embryos. (**d, f**) Each dot represents one biologically independent sample. (n=3 for Ctrl, n=4 for DomKD, and n=2 for DomB_KD, Rescue DomA, Rescue DomB each). **g**, Hatching rate of Ctrl and Hcf knockdown (HcfKD) embryos. (**e, g**)Each dot represents a biologically independent experiment of 50 embryos. Black lines represent the median of each condition. **h**, Expression of two H2A.Z positive genes (left and middle) and a Zld-dependent gene (right) in Ctrl and HcfKD ZGA embryos as measured by qPCR. Each dot represents one biologically independent sample. (n=4 for Ctrl, n=2 for DomKD, and n=2 for Hcf_KD). (**d, f, h**) Data are presented as mean values +/- SD. Expression is shown relative to rp49. **i**, Violin plot of the log2FoldChange in Pol II occupancy of ZGA embryos per transcript (non-redundant promoters, see Methods for details) in Zld-dependent, H2A.Z positive and H2A.Z negative genes. n represents the number of transcripts in each group. **j**, Violin plot of the log2FoldChange in GRO-Seq signal of ZGA embryos per gene (see Methods for details) in Zld-dependent, H2A.Z positive and H2A.Z negative genes. n represents the number of genes in each group. (**i, j**) H2A.Z negative genes were split into those that contain H2A.Z in the -1 position ('H2A.Z -1 nucleosome') and those that do not contain H2A.Z ('None'). All groups are statistically different by pairwise comparison (Wilcoxon rank sum test with continuity correction, two-sided, pvalue < 2.2e-16). **k**, Motif enrichment analysis of transcription factors in Zld-dependent (Zld-dep), H2A.Z positive (H2A.Z pos) and H2A.Z negative (H2A.Z neg) promoters. **l**, Gene set enrichment analysis using Metascape[6] showing the top 20 most significant GO terms per group (Zld-dep, H2A.Z pos and H2A.Z neg). Each GO-term is supported by the color scale in -log10 q-value and the fraction of observed genes presenting the proportion of observed genes in the respective GO term. Data representation was arranged as proposed by ref.[7]. **m**, Heatmap of Z-scores for gene expression over the first 24 hours of development for Zld-dependent, H2A.Z positive and H2A.Z negative genes. Developmental time in hours (h) is indicated on the bottom. Data taken from modENCODE[8]. **n**, Box plot of the variance of gene expression over developmental time for the data represented in (**m**). n = 2 for time points: 0-2, 2-4, 10-12, 12-14, 16-18, 18-20, 20-22, 22-24hrs, n=1 for time points 4-6, 6-8, 8-10, 14-16 hrs. **o**, Expression (log FPKM) during the first 10hours of development of genes classified as inactive at ZGA (NC14). Data taken from modENCODE[8]. Maternal genes (top) or non-maternal genes (bottom) were split by their H2A.Z content. 'H2A.Z_pos' contain H2A.Z on the +1-nucleosome position, while 'H2A.Z_neg' do not. 'Others' represent genes that could not be classified. n=2 for time points 0-2, 2-4 hrs, n=1: 4-6, 6-8, 8-10 hrs. Box plots (**c, i, j, n, o**) depict the median and the interquartile range (IQR) from the 1st to the 3rd quartile. Whiskers indicate the upper (Q3 + 1.5*IQR) and lower edge (Q1 - 1.5*IQR). (**d-h, k, n, o**) Source data are provided as a Source Data file.

Supplementary Figure 4

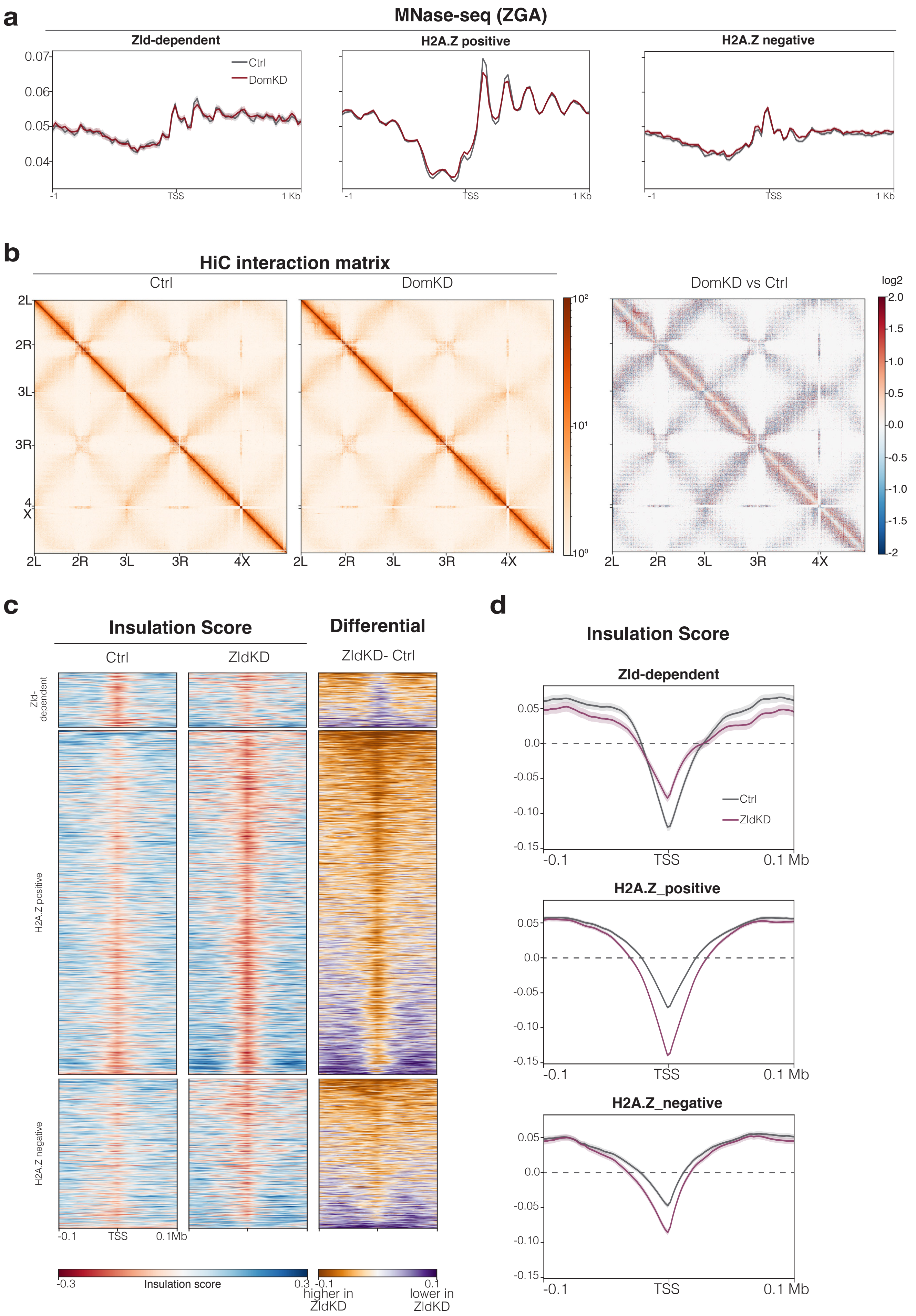

Supplementary Figure 4| Domino depletion does not affect nucleosome positioning but alters local chromatin structure, related to Fig.4. a, Profile plots of MNase-seq in Ctrl (grey) and DomKD (red) ZGA embryos in Zld-dependent, H2A.Z positive and H2A.Z negative genes. Corresponding to the heatmaps shown in Fig.4a. **b**, HiC interaction matrices of Ctrl and DomKD embryos. Differences on chromosome 2L are due to an inversion in the DomKD line that has no effect on phenotype. The matrix to the far right shows the ratio of contacts in the form of log2 (DomKD/Ctrl). **c**, Data from Hug et al., 2017[9]. Heatmap of the insulation score in Ctrl (left) and Zelda knockdown (ZldKD, middle) ZGA embryos sorted by their difference (right) and grouped in Zld-dependent, H2A.Z positive and H2A.Z negative. In the differential heatmap, purple colour represents lower insulation in the ZldKD embryos, while the orange signal represents higher insulation in the ZldKD embryos. **d**, Data from Hug et al., 2017[9]. Profile plots of insulation score in Zld-dependent, H2A.Z positive and H2A.Z negative promoters comparing Ctrl vs ZldKD ZGA embryos. For profile plots (**a,d**) the center line indicates the median and the semi-transparent lines represent the standard error of a given group and condition.

Supplementary Table 1. List of antibodies used for this study

PRIMARY ANTIBODIES					
**Antibody**	**Source/Company**	**Cat No.**	**lot No.**	**Assay**	**Usage**
H2A.Z *Drosophila*	Active Motif	39715	8614002	Western Blot	1:5000 dilution
H2A.Z *Drosophila*	Active Motif	39715	8614002	Immunofluorescence	1:100 dilution
H2A.Z *Drosophila*	Active Motif	39715	417003	ChIP-seq	4μl per sample
H2A.Z *Drosophila*	Active Motif	39715	8614002	RELACS ChIP-seq	4μl per sample
H3K4me3	Diagenode	C15410003-50	A1052D	ChIP-seq	1μg per sample
H3K36me3	Abcam	ab9050	GR273247-1	ChIP-seq	2μg per sample
H3K27ac	Diagenode	C15410196	A1723-0041D	ChIP-seq	1μg per sample
H3	Active Motif	39763	30615017	Immunofluorescence	1:500 dilution
lamin (Dm DL101s)	Developmental studies hybridoma bank (DSHB)	ADL-101	-	Western Blot	1:20000 dilution
GFP	Torrey Pines	TP401	81211	Immunofluorescence	1:500 dilution
Flag (M2)	Sigma	F1804	slbj4607v	Western Blot	1:2500 dilution
Tubulin-alpha, clone DM1A	Sigma	T9026	066M4870V	Western Blot	1:30000 dilution
H4K12ac	Merck, Upstate	07-595 Serum	3272292	Cut&Tag	1:75 (2μl in 150μl)
H4K12ac	Merck, Upstate	07-595 Serum	3272292	Immunofluorescence	1:2500 dilution
H4K12ac	Merck, Upstate	07-595 Serum	3272292	Western Blot	1:10000 dilution
Rpb3	Carla Margulies, Biomedical Center, LMU	-	-	ChIP-seq/ RELACS ChIP-seq	1:200 dilution
Zelda	Melissa Harrison, University of Wisconsin School of Medicine and Public Health	-	-	Cut&Tag	6μl per sample
phospho-H2A.Z	Developmental studies hybridoma bank (DSHB)	UNC93-5.2.1-s	-	Immunofluorescence	1:20 dilution
**SECONDARY ANTIBODIES**					
**Antibody**	**Company**	**Cat No.**	**lot No.**	**Assay**	**Usage**
Guinea Pig anti-Rabbit IgG	antibodies-online	Abin101961	43586-200-12190001	Cut&Tag	0.75 μg per sample (1:100 dilution)
Peroxidase goat anti mouse	Jackson ImmunoResearch	115-035-062	138817	Western Blot	1:5000 dilution
Peroxidase goat anti rabbit	Jackson ImmunoResearch	111-035-003	140987	Western Blot	1:5000 dilution
Alexa Fluor 488 F(ab')2 fragment	Molecular Probes by ThermoScientific	A11017	1812170	Immunofluorescence	1:500 dilution
Alexa Fluor 555 F(ab')2 fragment	Molecular Probes by ThermoScientific	A21425	1802486	Immunofluorescence	1:500 dilution
Alexa Fluor 488 F(ab')2 fragment	Molecular Probes by ThermoScientific	A11070	1775509	Immunofluorescence	1:500 dilution
Alexa Fluor 555 F(ab')2 fragment	Molecular Probes by ThermoScientific	A21430	2108786	Immunofluorescence	1:500 dilution

Supplementary Methods

Design and overexpression of hairpin resistant (rescue) domino isoforms. Hairpin resistant transgenes were designed by substituting the shRNA binding region for the corresponding region in the human homolog SRCAP. On top of this, all possible synonymous mutations were also introduced. This sequence is identical for the two domino isoforms.
Original sequence: tcagctgatacacgaagataa
Hairpin resistant sequence: cgaagttatcaatgacgagca

RNA-seq. For each genotype, four biological replicates were performed. RNA extraction was performed as in ref.[10]. Briefly, chloroform extraction was performed followed by RNA precipitation with Isopropanol. Samples were then washed twice with 80% EtOH and resuspended in TURBO DNase (Ambion AM2238) reaction mix. DNase was inactivated and for sequencing, RNA libraries were prepared using the Illumina TruSeq Stranded Total RNA protocol. Sequencing was done using the HiSeq, NextSeq or NovaSeq Illumina platform together with the paired end sequencing option. For qPCR, RNA (after DNase inactivation) was used for cDNA synthesis (First Strand cDNA Synthesis, ThermoScientific #K1612), followed by qPCR in a total reaction volume of 10μl (FastStart Universal SYBR Green Master (Rox) Roche 04913850001) with the subsequent program: Preincubation (600 sec at 95 °C), 2 Step Amplification (15 sec at 95 °C, 60 sec at 60 °C – repeat 55 times), Melting Curve (15 sec at 95 °C, 60 sec at 60 °C, 1 sec at 95 °C), Cooling (30 sec at 37 °C). Ct values were determined using the fitpoint method and then exported to further analyze the expression levels. The mRNA levels were normalized to the ribosomal gene Rp49.
The following primer pair sequences were used (5'to 3'):
dom(both isoforms): CAGACCGCGTAACTCCACATT and GCTGCTCCTACTGATCCTGC
domA specific: AAACAAGCGTGGGACGGGTA and CATGGGTGCGCAGATTTCCC
domB specific: ACCGCCCACTAGAAAGACAA and CGACGATCAAAGAGGCATCC
ihog: GCTGGAAACTCTGTGCTCTG and TCCGATGAACTCCGGTTTGA
gbb: TCAGCGATCAGGATGAGGAC and TTGTCCAGGTCGGTGATGAA
ac: GCAGGTCAACAATGGCTTCA and CCGGCGACCATTGCTTAAAT
Rp49: CTAAGCTGTCGCACAAATGG and GGGCATCAGATACTGTCCCT

Western Blot. Embryos were collected into Laemmli-Buffer (2 embryos per µl) and crushed with a pestle and subsequently run on SDS- PAGE and transferred to PVDF membrane using Wet-Blot. 24 unfertilized eggs or embryos were loaded per lane. For the chromatin fraction, 9µg and 3µg were loaded per lane. The ECL signal was recorded using the ChemiDoc Imaging System (Bio-Rad). Uncropped and unprocessed scans of all blots presented in this manuscript can be found in the Source Data file. For all antibodies used see Supplementary Table 1.

RNA-seq differential gene expression analysis. RNA-seq data was processed as defined in "ChIP-seq, RELACS ChIP-seq, ATAC-seq and RNA-seq data processing". Differential gene expression in RNA-seq was identified with *DEseq2-v1.2.6*[11] using the *snakePipes-v2.0.2*[12]. *featureCounts* table. Genes with less than 10 reads on average across replicates and samples were removed, and standard *DESeq2* workflow was executed using lfcShrinkage (*type = 'normal'*) and considering batches inside the *DESeq2* model matrix. Afterwards, only genes with TPM >5 in Ctrl or DomKD embryos were retained.

Timecourse analysis. Published RNA data of *Drosophila melanogaster* embryos from different developmental stages produced for the modENCODE project[8] (http://www.modencode.org/) was used. *Parallel-fastq-dump -v0.6.6* has been used to download the data. This data was processed using *snakePipes-v2.1.1*, trimmed and mapped on dm6. As part of the *snakePipes* output, a count matrix was generated which includes the reads' coverage on all dm6 genes (*ensembl-96*), for each developmental stage. Read counts were library-size normalized by using *DESeq2-v1.26.0* size factors, then replicates were averaged and log2 transformed. Next, for each gene, z-scores were computed. For visualization in Extended Data Fig.3o, inactive protein coding genes were selected and the time course matrix was reduced to cover 0-10 h for visualization using the hierarchical clustering implemented in the R-package *pheatmap-v1.0.12* (CRAN-link: https://cran.r-project.org/package=pheatmap).

Visualization of H2A.Z on enhancers. The list of enhancer candidates has been downloaded from https://enhancers.starklab.org/ based on ref.[1]. The coordinates were mapped to dm6 from dm3 using the *liftover* tool from *UCSCtools* toolbox[13]. Afterwards, *computeMatrix* on *scale-region* mode and *plotHeatmap* from *deeptools-v3.4.3*[14] were used consecutively to generate a matrix of H2Az coverage on these regions.

Supplementary References

1. Kvon, E. Z. *et al.* Genome-scale functional characterization of Drosophila developmental enhancers in vivo. *Nature* **512**, 91–95 (2014).
2. Li, Z. *et al.* Lipid droplets control the maternal histone supply of Drosophila embryos. *Curr. Biol.* **22**, 2104–2113 (2012).
3. Thorvaldsdóttir, H., Robinson, J. T. & Mesirov, J. P. Integrative Genomics Viewer (IGV): high-performance genomics data visualization and exploration. *Brief. Bioinform.* **14**, 178–192 (2013).
4. Lott, S. E. *et al.* Low concentration sequencing library loading protocol (Supplementary). *PLoS Biology* vol. 9 (2011).
5. Kwasnieski, J. C., Orr-Weaver, T. L. & Bartel, D. P. Early genome activation in Drosophila is extensive with an initial tendency for aborted transcripts and retained introns. *Genome Res.* **29**, 1188–1197 (2019).
6. Zhou, Y. *et al.* Metascape provides a biologist-oriented resource for the analysis of systems-level datasets. *Nat. Commun.* **10**, 1523 (2019).
7. Yu, G., Wang, L.-G., Han, Y. & He, Q.-Y. clusterProfiler: an R Package for Comparing Biological Themes Among Gene Clusters. *Omi. A J. Integr. Biol.* **16**, 284–287 (2012).
8. Celniker, S. E. *et al.* Unlocking the secrets of the genome. *Nature* **459**, 927–30 (2009).
9. Hug, C. B., Grimaldi, A. G., Kruse, K. & Vaquerizas, J. M. Chromatin Architecture Emerges during Zygotic Genome Activation Independent of Transcription. *Cell* **169**, 216-228.e19 (2017).
10. Zenk, F. *et al.* Germ line–inherited H3K27me3 restricts enhancer function during maternal-to-zygotic transition. *Science (80-.).* **357**, 212–216 (2017).
11. Love, M. I., Huber, W. & Anders, S. Moderated estimation of fold change and dispersion for RNA-seq data with DESeq2. *Genome Biol.* **15**, 550 (2014).
12. Bhardwaj, V. *et al.* snakePipes: facilitating flexible, scalable and integrative epigenomic analysis. *Bioinformatics* **35**, 4757–4759 (2019).
13. Kent, W. J., Zweig, A. S., Barber, G., Hinrichs, A. S. & Karolchik, D. BigWig and BigBed: Enabling browsing of large distributed datasets. *Bioinformatics* **26**, 2204–2207 (2010).
14. Ramírez, F. *et al.* deepTools2: a next generation web server for deep-sequencing data analysis. *Nucleic Acids Res.* **44**, W160–W165 (2016).
