# Supplementary material for: Histone variant H2A.Z regulates zygotic genome activation
Source: Nat Commun. 2021 Dec 1;12:7002. doi: 10.1038/s41467-021-27125-7 (PMC8636486; doi:10.1038/s41467-021-27125-7)
Supplement: Supplementary file 3 — Description of additional Supplementary File [file 41467_2021_27125_MOESM3_ESM.pdf]

Description of additional supplementary files

File Name: Supplementary Data 1
Description: Drosophila promoter classification according to transcriptional activity at ZGA.

File Name: Supplementary Data 2
Description: H2A.Z IPMS significantly enriched interactors over Control.

File name: Supplementary Data 3
Description: Differential analysis for GRO-seq, RNA-seq and Pol II occupancy in DomKD vs Ctrl embryos. Analysis of differential GRO-seq and RNA-seq was performed with DESeq2: two-sided, FDR corrected. Analysis of differential Poll II occupancy was performed with edgeR, two-sided, FDR corrected

File Name: Supplementary Data 4
Description: Gene expression of maternally deposited RNA
