# Supplementary material for: Histone variant H2A.Z regulates zygotic genome activation
Source: Nat Commun. 2021 Dec 1;12:7002. doi: 10.1038/s41467-021-27125-7 (PMC8636486; doi:10.1038/s41467-021-27125-7)
Supplement: Supplementary file 8 — Reporting Summary [file 41467_2021_27125_MOESM8_ESM.pdf]

nature research

Corresponding author(s): Nicola Iovino

Last updated by author(s): Oct 18, 2021

Reporting Summary

Nature Research wishes to improve the reproducibility of the work that we publish. This form provides structure for consistency and transparency in reporting. For further information on Nature Research policies, see our Editorial Policies and the Editorial Policy Checklist.

Statistics

For all statistical analyses, confirm that the following items are present in the figure legend, table legend, main text, or Methods section.

n/a	Confirmed	
☐	☒	The exact sample size (*n*) for each experimental group/condition, given as a discrete number and unit of measurement
☐	☒	A statement on whether measurements were taken from distinct samples or whether the same sample was measured repeatedly
☐	☒	The statistical test(s) used AND whether they are one- or two-sided<br>*Only common tests should be described solely by name; describe more complex techniques in the Methods section.*
☒	☐	A description of all covariates tested
☐	☒	A description of any assumptions or corrections, such as tests of normality and adjustment for multiple comparisons
☐	☒	A full description of the statistical parameters including central tendency (e.g. means) or other basic estimates (e.g. regression coefficient) AND variation (e.g. standard deviation) or associated estimates of uncertainty (e.g. confidence intervals)
☐	☒	For null hypothesis testing, the test statistic (e.g. *F*, *t*, *r*) with confidence intervals, effect sizes, degrees of freedom and *P* value noted<br>*Give P values as exact values whenever suitable.*
☒	☐	For Bayesian analysis, information on the choice of priors and Markov chain Monte Carlo settings
☒	☐	For hierarchical and complex designs, identification of the appropriate level for tests and full reporting of outcomes
☒	☐	Estimates of effect sizes (e.g. Cohen's *d*, Pearson's *r*), indicating how they were calculated

Our web collection on statistics for biologists contains articles on many of the points above.

Software and code

Policy information about availability of computer code

Data collection	parallel-fastq-dump -v0.6.6 has been used to download the ModENCODE data used for the gene expression over developmental time analysis. Western Blots were imaged using ChemiDoc Imaging System with Image-Lab(Bio-Rad), qPCR data was collected using LightCycler 96 and LightCycler 480 (Roche). Zen(Zeiss) and LAS AF (Leica) were used to record immunofluorescence data.
Data analysis	ChIP-seq, RELACS ChIP-seq, ATAC-seq and RNA-seq data processing:<br>snakePipes/2.0.2<br><br>Blacklisting of hyper-accessible regions:<br>snakePipes/2.0.2<br><br>GRO-seq data processing and analysis:<br>umi_tools/1.0.0<br>cutadapt/2.5<br>bowtie2/2.3.3.1<br>samtools/1.10<br>deeptools/3.3.1<br>MultiQC/1.8<br>FastQC/0.11.5<br>subread/1.5.3<br><br>GRO-seq active genes:<br>macs2/2.1.2

sambamba/0.7.0

GRO-seq differential gene expression analysis:
DESeq2/1.26
subread/1.5.3

H2A.Z RELACS quantification:
deeptools/3.4.1
subread/1.5.3
DESeq2/1.26

Cut&Tag/Run processing and quantification:
snakePipes/2.4.3
deeptools/3.5.0
DESeq2/1.26

RNA-seq differential gene expression analysis:
DESeq2/1.26
snakePipes/2.0.2

Classification of H2A.Z positive promoters:
samtools/1.10
NucHunter/only version available

Zld-dependent promoters identification and Differential Pol II occupancy analysis:
featureCounts/
edgeR/3.28.1
subread/1.5.3

Motif Enrichment analysis:
MEME suite/5.0.2

GO analysis:
Metascape(https://metascape.org)/online version

Mass Spectrometry:
MaxQuant/1.6.14.0
R/4.0.3
limma/3.44.3
RStudio/1.3.1093-1

MNase data analysis:
snakepipes/2.1.0
deeptools/3.4.3

HiC data analysis:
snakePipes/2.1.0
hicexplorer/3.4.3
bedGraphToBigWig/4

Time course:
snakePipes/2.1.1
DESeq2/1.26.0
parallel-fastq-dump/0.6.6
pheatmap/1.0.12

Visualisation H2A.Z on enhancers:
lift over/UCSCtools toolbox
deeptools/3.4.3

General data analysis:
R/3.6.3
ggplot2/3.28.1
bedtools/2.27.0

For manuscripts utilizing custom algorithms or software that are central to the research but not yet described in published literature, software must be made available to editors and reviewers. We strongly encourage code deposition in a community repository (e.g. GitHub). See the Nature Research guidelines for submitting code & software for further information.

Data

Policy information about availability of data

All manuscripts must include a data availability statement. This statement should provide the following information, where applicable:

- Accession codes, unique identifiers, or web links for publicly available datasets
- A list of figures that have associated raw data
- A description of any restrictions on data availability

The sequencing data generated in this study have been deposited in the NCBI's Gene Expression Omnibus and are accessible through GEO Series accession number GSE173240 [https://www.ncbi.nlm.nih.gov/geo/query/acc.cgi?acc= GSE173240]. The mass spectrometry proteomics data generated in this study have been deposited to the ProteomeXchange Consortium via the PRIDE partner repository with the dataset identifier PXD029061 [http://proteomecentral.proteomexchange.org/cgi/GetDataset?ID=PXD029061]. Source data are provided with this paper, which includes raw data for graphs and gel blot images for Figs. 2d, 3c,3d, Sup. Figs. 2d-f, 3d-h, 3k, 3n and 3o in the Source Data file. Promoter classification for Fig.1 and Sup. Fig.1, and analysis files for Figs.2b, 3a,3b, Sup. Fig. 3c, 3i, 3j, are provided as Supplementary Data. MNase-seq data presented in Fig.1a and Fig.1c was obtained from ref.32, and is accessible through GEO accession numbers: GSM3736319 [https://www.ncbi.nlm.nih.gov/geo/query/acc.cgi?acc=GSM3736319], GSM3736320 [https://www.ncbi.nlm.nih.gov/geo/query/acc.cgi?acc=GSM3736320], GSM3736321 [https://www.ncbi.nlm.nih.gov/geo/query/acc.cgi?acc=GSM3736321], GSM3736322 [https://www.ncbi.nlm.nih.gov/geo/query/acc.cgi?acc=GSM3736322], GSM3736323 [https://www.ncbi.nlm.nih.gov/geo/query/acc.cgi?acc=GSM3736323], GSM3736324 [https://www.ncbi.nlm.nih.gov/geo/query/acc.cgi?acc=GSM3736324], GSM3736325 [https://www.ncbi.nlm.nih.gov/geo/query/acc.cgi?acc=GSM3736325]. RNA-seq data presented in Sup. Fig.3m-o was obtained from ref. 82 and is accessible through SRA: SRP001696 [https://trace.ncbi.nlm.nih.gov/Traces/sra/?study=SRP001696]. Specific samples used for this study have the accession numbers SRR1197327 to SRR1197338 and SRR1197363 to SRR1197370. HiC data presented in Sup. Fig.4c-d was obtained from ref. 33. and is accessible through ArrayExpress: E-MTAB-4918 [https://www.ebi.ac.uk/arrayexpress/experiments/E-MTAB-4918/] Specific samples used for this study have the accession numbers ERR1533226 to ERR1533236, and ERR1912884 to ERR1912887. Enhancer candidates used in Sup. Fig.1b were downloaded from https://enhancers.starklab.org/ based on ref. 61. All other data are available within the article and its Supplementary Information. Custom code for sequencing data analysis is accessible through the GitHub repository: https://github.com/iovinolab/dom-study_2020.

Field-specific reporting

Please select the one below that is the best fit for your research. If you are not sure, read the appropriate sections before making your selection.

- [x] Life sciences
- [ ] Behavioural & social sciences
- [ ] Ecological, evolutionary & environmental sciences

For a reference copy of the document with all sections, see nature.com/documents/nr-reporting-summary-flat.pdf

Life sciences study design

All studies must disclose on these points even when the disclosure is negative.

Sample size	All ChIP-Seq and HiC experiments have been performed in biological replicates. For RNA-Seq 3-4 biological replicates were collected. For GRO-Seq, 3 biological replicates were collected. We did not apply statistical methods to pre-determine sample size and followed the general standard practice in the field. The number of replicates in experiments is also stated in the legends.
Data exclusions	We did not exclude data.
Replication	HiC replicate correlation was assessed by verifying the QC of the pipeline, distance vs. counts and visualizing matrices (HiC pipeline of snakepipes-v2.1.0). MNase-seq replicate correlation was assessed by verifying the QC (DNA-mapping pipeline of snakepipes-v2.1.0). ChIP-seq, RELACS ChIP-Seq, ATAC-seq, GRO-seq and RNA-seq replicate correlation was assessed using deeptools correlation plots. We observed agreement between all the replicates, which were performed with biologically independent samples. HiC, MNase-seq, ChIP-seq, RELACS ChIP-seq and ATAC-seq experiments were performed with 2 biologically independent samples. RNA-seq was performed with 3-4 biologically independent samples, GRO-seq was performed with 3 biologically independent samples.
Randomization	No experiments that required randomization of the samples were performed. We controlled variability by collecting biologically independent samples in several batches. Embryos from the same developmental stage were collected in pools.
Blinding	No experiments that required blinding of groups were performed. Blinding was not possible given the noticeable differences in phenotype between the knockdown embryos and the controls.

Reporting for specific materials, systems and methods

We require information from authors about some types of materials, experimental systems and methods used in many studies. Here, indicate whether each material, system or method listed is relevant to your study. If you are not sure if a list item applies to your research, read the appropriate section before selecting a response.

Materials & experimental systems

n/a	Involved in the study
☐	☒ Antibodies
☒	☐ Eukaryotic cell lines
☒	☐ Palaeontology and archaeology
☐	☒ Animals and other organisms
☒	☐ Human research participants
☒	☐ Clinical data
☒	☐ Dual use research of concern

Methods

n/a	Involved in the study
☐	☒ ChIP-seq
☒	☐ Flow cytometry
☒	☐ MRI-based neuroimaging

Antibodies

Antibodies used

A description of all antibodies and dilutions used can be found in Supplementary Table1.
Histone H2Av antibody (Active Motif, Cat. No. 39715, lot No.00417003)
Histone H2Av antibody (Active Motif, Cat. No. 39715, lot No.8614002)
H3(Active Motif, Cat. No. 39763, lot No. 30615017)
H3K4me3 (Diagenode, Cat. No. C15410003-50)
H3K36me3 (Abcam, Cat. No. ab9050)
H3K27ac(Diagenode, Cat. No.C15410196, lot No.A1723-0041D)
lamin Dm DL101s (Developmental Studies Hybridoma Bank, Cat No., ADL-101)
GFP (Torrey Pines, Cat. No. TP401, lot No. 81211)
Flag (M2) (Sigma, Cat. No. F1804, lot No. slbj4607v)
Tubulin-alpha (Sigma, clone DM1A, Cat. No. T9026, lot No. 066M4870V)
H4K12ac (Merck, Upstate, Cat. No. 07-595 Serum, lot No. 3272292)
Rpb3 (Kindly provided by Carla Margulies, not commercial, and published)
Zelda (Kindly provided by Melissa Harrison, not commercial, and published)

Validation

Histone H2Av antibody manufacturer's statement: "This antibody has been validated for use in ChIP and/or ChIP-Seq" validation on the manufacturer's website: (https://www.activemotif.com/catalog/details/39715/histone-h2av-antibody-pab)
Rpb3 antibody was a gift from Carla Margulies and has been published (Schauer et al., 2013)
H3K4me3 antibody validation on the manufacturer's site (https://www.diagenode.com/en/p/h3k4me3-polyclonal-antibody-premium-50-ug-50-ul)
H3K36me3 antibody validation on the manufacturer's site (https://www.abcam.com/histone-h3-tri-methyl-k36-antibody-chip-grade-ab9050.html)
Tubulin-alpha antibody validation on the manufacturer's site (https://www.sigmaaldrich.com/catalog/product/sigma/t9026?lang=de&region=DE)
H4K12ac manufacturer's statement: "Use Anti-acetyl-Histone H4 (Lys12) Antibody (Rabbit Polyclonal Antibody) validated in ChIP, DB, WB, ChIP-seq to detect acetyl-Histone H4 (Lys12) also known as H4K12Ac, Histone H4 (acetyl K12)." (https://www.merckmillipore.com/DE/de/product/Anti-acetyl-Histone-H4-Lys12-Antibody,MM_NF-07-595?ReferrerURL=https%3A%2F%2Fwww.google.com%2F) The use of this antibody in Drosophila has been published (Scacchetti et al., 2020).
lamin Dm DL101s antibody validation on the manufacturer's site (https://dshb.biology.uiowa.edu/ADL101)
Flag M2 antibody validation on the manufacturer's site (https://www.sigmaaldrich.com/catalog/product/sigma/f1804?lang=de&region=DE)
Zelda antibody was a gift from Melissa Harrison and has been published (Harrison, MM et al., 2011)

Animals and other organisms

Policy information about studies involving animals; ARRIVE guidelines recommended for reporting animal research

Laboratory animals

Drosophila melanogaster. Fly stocks used in this study: TRiP line control (BDSC#36303), Domino shRNA (BDSC #41674), DominoB shRNA (BDSC #55917), Hcf shRNA (BDSC #36799), pUASp H2Av::FlagHA (This study), mat-alpha-Gal4 (BDSC #7062), DomA-GFP-Flag (kindly provided by Peter Becker, LMU Munich), DomB-GFP-Flag (kindly provided by Peter Becker, LMU Munich), deltaDom-GFP (kindly provided by Peter Becker, LMU Munich), DomB-Flag-HA (Rescue DomB,this study), UASp-DomA-Flag-HA (Rescue DomA, this study).

Wild animals

This study did not involve the use of wild animals

Field-collected samples

This study did not involve samples collected from the field

Ethics oversight

All work was conducted in Drosophila melanogaster, an invertebrate animal (hexapod arthropod from the insect group). Invertebrate model are not regulated by the TierSchVersV and therefore are not subjected to ethical approval.

Note that full information on the approval of the study protocol must also be provided in the manuscript.

ChIP-seq

Data deposition

☒ Confirm that both raw and final processed data have been deposited in a public database such as GEO.

☒ Confirm that you have deposited or provided access to graph files (e.g. BED files) for the called peaks.

Data access links<br>*May remain private before publication.*	ChIP-seq data generated for this study are deposited in a public database (GEO accession GSE161594)
Files in database submission	Raw sequencing files:<br>20200207_11_DI_Input_dm_T180_emb_rep1_c9-13_R1.fastq.gz<br>20200207_11_DI_Input_dm_T180_emb_rep1_c9-13_R2.fastq.gz<br>20200207_12_DI_ChIP-H2Av_dm_T180_emb_rep1_c9-13_R1.fastq.gz<br>20200207_12_DI_ChIP-H2Av_dm_T180_emb_rep1_c9-13_R2.fastq.gz<br>20200207_13_DI_ChIP-Rpb3_dm_T180_emb_rep1_c9-13_R1.fastq.gz<br>20200207_13_DI_ChIP-Rpb3_dm_T180_emb_rep1_c9-13_R2.fastq.gz<br>20200207_14_DI_Input_dm_T180_emb_rep2_c9-13_R1.fastq.gz<br>20200207_14_DI_Input_dm_T180_emb_rep2_c9-13_R2.fastq.gz<br>20200207_15_DI_ChIP-H2Av_dm_T180_emb_rep2_c9-13_R1.fastq.gz<br>20200207_15_DI_ChIP-H2Av_dm_T180_emb_rep2_c9-13_R2.fastq.gz<br>20200207_16_DI_ChIP-Rpb3_dm_T180_emb_rep2_c9-13_R1.fastq.gz<br>20200207_16_DI_ChIP-Rpb3_dm_T180_emb_rep2_c9-13_R2.fastq.gz<br>20200403_01_DI_ChIP_Rpb3_dm_T180xNi173_emb_rep1_st5_R1.fastq.gz<br>20200403_01_DI_ChIP_Rpb3_dm_T180xNi173_emb_rep1_st5_R2.fastq.gz<br>20200403_02_DI_ChIP_Rpb3_dm_T180xNi173_emb_rep2_st5_R1.fastq.gz<br>20200403_02_DI_ChIP_Rpb3_dm_T180xNi173_emb_rep2_st5_R2.fastq.gz<br>20200403_03_DI_ChIP_Rpb3_dm_T216xNi173_emb_rep1_st5_R1.fastq.gz<br>20200403_03_DI_ChIP_Rpb3_dm_T216xNi173_emb_rep1_st5_R2.fastq.gz<br>20200403_04_DI_ChIP_Rpb3_dm_T216xNi173_emb_rep2_st5_R1.fastq.gz<br>20200403_04_DI_ChIP_Rpb3_dm_T216xNi173_emb_rep2_st5_R2.fastq.gz<br>20200403_05_DI_Input_dm_T180xNi173_emb_rep1_st5_R1.fastq.gz<br>20200403_05_DI_Input_dm_T180xNi173_emb_rep1_st5_R2.fastq.gz<br>20200403_06_DI_Input_dm_T180xNi173_emb_rep2_st5_R1.fastq.gz<br>20200403_06_DI_Input_dm_T180xNi173_emb_rep2_st5_R2.fastq.gz<br>20200403_07_DI_Input_dm_T216xNi173_emb_rep1_st5_R1.fastq.gz<br>20200403_07_DI_Input_dm_T216xNi173_emb_rep1_st5_R2.fastq.gz<br>20200403_08_DI_Input_dm_T216xNi173_emb_rep2_st5_R1.fastq.gz<br>20200403_08_DI_Input_dm_T216xNi173_emb_rep2_st5_R2.fastq.gz<br>527_1_180_c14_H3K36me3_new_ab_rep1_6_R1.fastq.gz<br>527_1_180_c14_H3K36me3_new_ab_rep1_6_R2.fastq.gz<br>527_1_180_c14_H3K4me3_rep1_8_R1.fastq.gz<br>527_1_180_c14_H3K4me3_rep1_8_R2.fastq.gz<br>527_1_180_c14_input_rep1_1_R1.fastq.gz<br>527_1_180_c14_input_rep1_1_R2.fastq.gz<br>527_2_180_c14_H3K36me3_new_ab_rep2_10_R1.fastq.gz<br>527_2_180_c14_H3K36me3_new_ab_rep2_10_R2.fastq.gz<br>527_2_180_c14_H3K4me3_rep2_12_R1.fastq.gz<br>527_2_180_c14_H3K4me3_rep2_12_R2.fastq.gz<br>527_2_180_c14_input_rep2_2_R1.fastq.gz<br>527_2_180_c14_input_rep2_2_R2.fastq.gz<br>Input1_T180xNI173_St5_R1.fastq.gz<br>Input1_T180xNI173_St5_R2.fastq.gz<br>Input3_T216xNI173_St5_R1.fastq.gz<br>Input3_T216xNI173_St5_R2.fastq.gz<br>Input4_T180xNI173_St5_R2_R1.fastq.gz<br>Input4_T180xNI173_St5_R2_R2.fastq.gz<br>Input6_T216xNI173_St5_R2_R1.fastq.gz<br>Input6_T216xNI173_St5_R2_R2.fastq.gz<br>IP1_H2Av_T180xNI173_St5_R1.fastq.gz<br>IP1_H2Av_T180xNI173_St5_R2.fastq.gz<br>IP11_H2Av_T216xNI173_St5_R2_R1.fastq.gz<br>IP11_H2Av_T216xNI173_St5_R2_R2.fastq.gz<br>IP5_H2Av_T216xNI173_St5_R1.fastq.gz<br>IP5_H2Av_T216xNI173_St5_R2.fastq.gz<br>IP7_H2Av_T180xNI173_St5_R2_R1.fastq.gz<br>IP7_H2Av_T180xNI173_St5_R2_R2.fastq.gz

Processed files:
H2Av.subtract_input.ctrl.c9-13.pooled.bw
H2Av.subtract_input.ctrl.pooled.bw
H2Av.subtract_input.dom-kd.pooled.bw
h3k36me3.subtract_input.ctrl.mean.bw
h3k4me3.subtract_input.ctrl.mean.bw
Pol2.subtract_input.ctrl.pooled.bw
Pol2.subtract_input.dom-kd.pooled.bw
Rpb3.ctrl.c9-13.pooled.bw
Pol2_ChIP.dom-kd.dm6_ensembl96.transcripts.tsv

Genome browser session (e.g. UCSC)

No longer applicable

Methodology

Replicates

Two biological replicates were performed for each ChIP-seq experiment.

Sequencing depth

We sequenced all samples in the study at least to a depth of 15 Mio.

Antibodies

Histone H2Av antibody (Active Motif, Cat. No. 39715, lot No.00417003)
Rpb3 (Kindly provided by Carla Margulies)
H3K4me3 (Diagenode, Cat. No. C15410003-50)
H3K36me3 (Abcam, Cat. No. ab9050)
Histone H2Av antibody (Active Motif, Cat. No. 39715, lot No.8614002)

Peak calling parameters

No Peak calling was performed in any of the ChIP-seq experiments

Data quality

No Peak calling was performed in any of the ChIP-seq experiments

Software

ChIP-seq, RELACS ChIP-seq, ATAC-seq and RNA-seq data processing:
Raw short read sequencing was processed uniformly using default parameters of snakepipes-v2.0.2 (Bhardwaj et al., 2019). RNA-seq was processed using the mRNA-seq workflow (–trim option). ChIP-seq, RELACS and ATAC-seq were processed with the DNA-mapping workflow (–trim, --dedup, –properPairs), followed by assay-specific workflow, i.e. ChIP-seq and RELACS with the ChIP-seq workflow and ATAC-seq by the ATAC-seq workflow. All libraries were mapped to dm6, using the Ensembl version 96 annotation (Yates et al., 2019).

H2A.Z RELACS quantification:
The quantification H2A.Z was performed a) using the local scaling factors as defined in Arrigoni et al., 2018, with the focus on active promoters (+/- 400 nt around TSS) and b) using the DEseq2 rlog transformation. For the local scaling factors, deeptools' multiBamSummary was use to count paired sequence reads in the defined promoters (multiBamSummary BED-file –extendReads ,Ramírez et al., 2016) followed by the library-size corrected double ratios of IP and Input as described previously (Arrigoni et al., 2018). For the promoter quantification of H2Av RELACS IP and Input, reads per promoter were counted using featureCounts (-t promoter -g transcript_id -f -Q 3 --primary -s 0 -p -B, Liao et al., 2014). Then, for the quantification, DEseq2 was used to compute the rlog transformation of the read counts. Since RELACS is quantitative, the size factors for library size correction were computed from the input only. Finally, rlog values were averaged between replicates.

Identification and classification of H2A.Z positive promoters:
In order to identify promoters enriched for H2A.Z, NucHunter (Mammana et al., 2013) was used to predict nucleosome positions from the H2A.Z ChIP-seq data on wild type samples. In order to do that, alignments were filtered for their quality using 'samtools view -q 3' from samtools-v1.10 (Li et al., 2009). Fragment length of the filtered alignments were predicted using fraglen tool of NucHunter, these fragment lengths were used to run callnucs tool of NucHunter to predict the position of nucleosomes. Plus 1 nucleosomes (+1) were then predicted using the following criteria: 1) A nucleosome within 350 nucleotides from a TSS was predicted for at least one H2A.Z ChIP-seq replicate or 2) A nucleosome within 600 nucleotides from a TSS was predicted in both replicates, with less than 80 nucleotides difference from the center of the predicted nucleosomes between the two replicates. A promoter was considered H2A.Z positive, if there was a predicted +1 nucleosome overlapping or downstream the same promoter. The -1 nucleosomes were identified in the same way, but scanning upstream of the TSS.

Zld-dependent promoters identification and Differential Pol II occupancy analysis:
Differential Pol II occupancy was determined using the method described in Blythe and Wieschaus, 2015. In brief, reads were countered per transcript using featureCounts (-t transcript -g transcript_id -f -O -Q 3 --primary -s 0). Reads were counted multiple times if they overlap several isoforms. Then, per transcript read counts were processed with edgeR (Robinson et al., 2010) to compute differential Pol2 occupancy. Like in the published method, transcripts spanning less than 125 nucleotides were discarded and only transcripts with read counts across replicates sum more that 10 reads were considered.
This method was used to identify Zelda targets in dm6 (logFC < -1 and FDR < 0.01) using published Pol II ChIP-seq data in Zld mutants (Blythe and Wieschaus, 2015). Pol II occupancy per transcript was quantified using edgeR's FPKM calculations.
This same method was also used to quantify Pol II in DomKD and Ctrl ZGA embryos, with the following modifications: First, edgeR norm.factors were computed on our Zelda zygotic targets (see "Maternal/Zygotic classification and Zelda zygotic target definition" in

the manuscript, Methods section). Differential transcripts were identified using the likelihood ratio test (edgeR::glmLRT). Then, unique promoters were identified and selected based on the most significant change among all transcripts for a given promoter (min FDR, edgeR results).
